# Weight Pressures and Eating Disorder Symptoms among Adolescent Female Gymnasts of Different Performance Levels in Greece

**DOI:** 10.3390/children9020254

**Published:** 2022-02-14

**Authors:** Ioanna Kontele, Tonia Vassilakou, Olyvia Donti

**Affiliations:** 1 Department of Public Health Policy, School of Public Health, University of West Attica, 196 Alexandras Avenue, 11521 Athens, Greece; tvasilakou@uniwa.gr; 2 School of Physical Education and Sport Science, National Kapodistrian University of Athens, 17237 Athens, Greece

**Keywords:** adolescence, gymnastics, diet, disordered eating, appearance, performance

## Abstract

This study examined the weight pressures within the gymnastics environment and explored associations between these pressures and eating disorder (ED) symptoms in adolescent female gymnasts. One hundred and forty-seven competitive gymnasts and 122 recreational-level gymnasts (11–17 years old) completed the Eating Attitudes Test (EAT-26), the Weight-Pressures in Sport-Females (WPS-F), the Social Desirability Scale (SDS) and provided information on their training. It was found that 16.3% of competitive gymnasts and 7.4% of non-competitive gymnasts scored ≥20 in EAT-26, indicating disordered eating behavior. Competitive gymnasts scored higher than non-competitive in the total score of EAT-26 (*p* = 0.027), as well as in the total score of WPS-F, the sport and coaches weight pressures sub-scale and the appearance and performance weight pressures sub-scale (*p* < 0.001). Multiple regression analyses indicated that sport and coaches weight pressures, appearance and performance weight pressures and body mass index accounted for 30.3% of the variance of EAT-26 in competitive gymnasts, while the appearance and performance weight pressures sub-scale accounted for 16.3% of the variance of EAT-26 in non-competitive gymnasts. Sport and coaches weight pressures are associated with ED in competitive gymnasts, while body appearance and performance demands correlate with ED in female gymnasts irrespective of competitive level.

## 1. Introduction

Gymnastics sports are popular among female adolescents and include seven different disciplines: Artistic Gymnastics, Rhythmic Gymnastics, Trampoline, Tumbling, Acrobatic Gymnastics, Aerobic Gymnastics and Gymnastics for All [1,2]. Female gymnasts start intensive training from childhood and reach peak performance level in adolescence, typically training for 4–6 h per day in order to compete at international level [3].

Gymnastics is a weight-sensitive and a weight-bearing sport because gymnasts must lift their own body weight against gravity. All women gymnastics disciplines require specific performance characteristics, such as strength, flexibility, balance and movement precision [4]. Low body weight and especially low fat mass, combined with muscle strength and power, are considered beneficial for technique because they increase the power-to-weight ratio. This is critical for optimal gymnastics performance since athletes must rapidly transfer their body mass across space while overcoming gravitational resistance [3,5,6]. Respectively, excessive fat mass is a disadvantage, as it decreases the efficiency of movements [7]. A number of studies have demonstrated correlations between fat mass and athletes’ performance. Claessens et al. (1999) suggested that gymnasts with more subcutaneous fat have lower performance scores [8]. Moreover, Sherman et al. (1996) found that world-class female gymnasts with relatively moderate BMI tended to outperform athletes with particularly high or particularly low BMI [9]. In addition, lean body shape is considered important for sport-specific aesthetic reasons [8]. Research in gymnastics sports has demonstrated that short stature, low body mass and low body fat are characteristics of elite female artistic gymnasts and that low body weight, low body fat, and higher than average height are characteristics of elite rhythmic gymnasts [3,5,10,11]. Moreover, in acrobatic gymnastics, athletes of “top” position have low body weight and fat mass [12]. Collectively, low body weight and fat mass are important for gymnastics performance irrespective of the discipline.

Many gymnasts, in their quest for the ideal body weight or shape, often follow low calorie diets and other unhealthy weight-control practices [13,14,15,16,17,18]. As a result, many female adolescent gymnasts do not meet the nutritional requirements for energy, macronutrients and micronutrients, and they may be at risk of low energy availability and nutritional deficiencies [19,20,21,22,23,24,25,26,27]. Moreover, the use of unhealthy weight control practices puts female gymnasts at a high risk of developing eating disorders (ED) and disordered eating (DE) [28,29,30,31,32,33].

Eating disorders (ED) are serious mental illnesses that meet clinical criteria described in the Diagnostic and Statistical Manual of Mental Disorders of the American Psychiatric Association (DSM-5) [34]. In athletic populations, disordered eating (DE) is more common than ED and refers to the use of unhealthy weight-control behaviors (e.g., skipping meals, fasting, overexercising, vomiting, using diuretics, etc.) without meeting the criteria for clinical diagnosis of an eating disorder [35,36]. The onset of ED is typically observed during adolescence, with the peak age of anorexia nervosa starting in early-to mid-adolescence and of bulimia nervosa in late adolescence [37]. Eating disorders and disordered eating have been associated with serious health problems in adolescence [38]. Adolescent athletes seem to be at higher risk, as their body struggles to meet the simultaneous demands of growth, maturation and intensive training [39,40]. Low energy availability, macronutrient and micronutrient deficits, dehydration, electrolyte imbalances and low bone mass density are among the consequences of ED or DE in adolescent athletes [41]. Low energy availability is also considered the main reason for the development of the Female Athlete Triad, as it disrupts the normal cycle of menstruation and may cause an imbalance in bone remodeling [42].

Research indicates that ED symptomatology may reach 5% to 45% in female athletes [13,14,29,30,31,32] and is higher in athletes than non-athletes [28,43] and in lean sports than non-lean sports [44]. However, the influence of competition level on ED risk is less studied and the results are ambiguous. Kong and Harris (2015), in a study with 320 female athletes, found that elite female athletes had higher ED prevalence compared to recreational and non-competitive athletes [45]. Along this line, Francisco et al. (2013) found that elite female athletes demonstrated greater risk of developing eating disorders than non-elite athletes [46]. Recently, Donti et al. (2021) reported a higher prevalence of eating disorder symptoms in international compared to recreational-level rhythmic gymnasts [32]. In contrast, ED symptomatology did not differ between elite and non-elite female skaters in the USA [47], in a study of 846 female athletes from 67 different sports in Finland [48] or in a study of athletes from a broad range of sports in Spain [49].

The etiology of eating disorders is multifactorial. Genetic and environmental factors are often identified [50]. High competitiveness, early sport-specialization, requirement of a specific weight to compete in a specific category and pressures from the sport environment to achieve a certain body weight or shape are considered sport-related risk factors for the development of ED in athletes [50]. Among the above-mentioned factors, pressure to achieve or keep a low body weight and/or a lean body shape is considered important for the development of ED [51,52,53]. The most common weight-related pressures from the sports environment are the comments that may come from coaches, teammates and judges about weight and body shape of an athlete, the requirements for a specific weight to compete in a specific category, regular weightings, the perception that low weight is associated with performance advantages and the training or competition uniform that may reveal bodily flaws [54]. Different types of sport environment weight pressures have been associated with ED symptomatology. Coaches’ negative comments about weight and shape and the pressure they put on athletes to attain an ideal weight seem to be one of the most powerful factors associated with ED in female athletes, including gymnasts [45,49,51,55,56]. Comments regarding weight may also come from judges and teammates [57,58]. Moreover, the belief that low body weight is a prerequisite in order to improve sports performance has been reported in studies of dancers, cheerleaders, swimmers and athletes of various aesthetic sports [17,59,60,61,62]. Finally, a number of studies in cheerleaders, swimmers and dancers have identified the competition uniform as a factor associated with weight pressures, especially if this has a form that reveals perceived bodily flaws [49,59,61,63].

In Greece, research has indicated that the prevalence of eating disorder symptoms in female athletic population ranges from 11% to 46,3% [31,32,64,65,66]. The factors that have been related to the development of ED in Greek athletes are anxiety, perfectionism and disturbed body image, as well as training experience, competition level and BMI [31,32,65,67,68]. However, to date, no study in Greece has investigated the weight pressures that female athletes experience in the sports environment, nor the potential association between weight pressures and the development of ED symptoms. The aim of the present study was to address this gap by examining the weight pressures that come from the gymnastics environment and to explore potential correlations between those pressures and eating disorder symptoms in a sample of competitive and non-competitive adolescent female gymnasts. It was hypothesized that: (a) a higher percentage of ED symptoms would be observed in competitive gymnasts than in non-competitive gymnasts, (b) ED symptoms would be associated with weight pressures that come from the sport environment and (c) the level of perceived weight pressures would be higher as the competition level increases.

## 2. Materials and Methods

### 2.1. Participants’ Recruitment and Inclusion Criteria

Data collection took place between August and November 2020. At first, researchers contacted the coaches of 77 gymnastics clubs, as well as the coaches of the two national training centers, through an official invitation published in the national gymnastics federation’s website, through email and through personal contacts.

Coaches of 27 regional gymnastics clubs (35% of the total clubs invited) from Athens, Thessaloniki, Kavala and Crete, as well as coaches from the two national training centers in Athens and Thessaloniki, agreed to participate in this research. In order to be included in the study, athletes had to meet the following criteria: (a) being female, (b) being 11–17 years old, (c) training in one of the disciplines of gymnastics (i.e., artistic, rhythmic, trampoline, tumbling, acrobatics, aerobics or gymnastics for all), (d) providing personal and parental/guardian consent. All athletes from the participating clubs who met the above-mentioned criteria were eligible to join the study. Athletes were excluded if they (a) were males, (b) were younger than 11 or older than 17 years, (c) did not provide parental consent or (d) their parents reported that the child had a nutrition-related problem (such as diabetes, celiac disease, anorexia nervosa, etc.).

In this study, only female gymnasts were included. This was decided due to the fact that this sport attracts mainly girls, and also that female gymnasts are considered to face higher pressures to maintain a low body weight, according to previous studies.

For the purposes of this study, athletes were divided into two groups: (a) competitive and (b) non-competitive. All gymnasts of artistic, rhythmic, trampoline, tumbling, aerobic and acrobatic gymnastics that competed in national or/and international competitions were considered competitive athletes. Athletes of the above-mentioned disciplines that declared participation only to local and regional events or gymnastics festivals were considered as non-competitive gymnasts. Moreover, all the athletes of “gymnastics for all” discipline were included in the non-competitive group, as this discipline demands only participation in recreational and contest events and not competitions.

### 2.2. Ethical Permission, Consent and Anonymity

This study was a part of the project “Eating habits, weight pressures and disordered eating attitudes of adolescent female athletes of gymnastics in Greece”. The study has received the approval of the Ethics Committee of the University of West Attica (reference number 52760/date 21 July 2020) and the Hellenic Gymnastics Federation (reference number 3151/date 28 August 2020).

A parent or legal guardian’s informed consent was obtained for each athlete. Parents and guardians were informed of the purpose of the study, the confidentiality of the data and the anonymity of the athletes. The contact information of the main researcher for further questions was also provided. After collecting the parents’ consent, researchers visited the training facilities, with the permission of the coaches, and distributed the questionnaires to the athletes before training. The researchers provided written information and they also orally explained to the athletes that there were no right or wrong answers, that it was important to give accurate answers and that all their data were anonymous. Moreover, it was explained that athletes were free to withdraw whenever they wanted.

### 2.3. Questionnaires and Tools applied

#### 2.3.1. Eating Attitudes Test-26

Eating Attitudes Test (EAT-26) was used to assess eating disorder symptoms. EAT-26 is a self-report questionnaire consisting of 26 questions with 6 possible answers. The score of each question ranges from 0 to 3 (3 = often, 2 = usually, 1 = always, 0 = sometimes, rarely, never), and the final score of the questionnaire ranges from 0–78. An overall score of ≥20 is indicative of disordered eating attitudes. The questionnaire consists of three sub-scales: (a) Dieting (being preoccupied with thinness and avoiding fattening foods), (b) Bulimia and Food Preoccupation (thinking about food and bulimic symptomatology) and (c) Oral Control (self-control of eating and perceived pressures from others to gain weight) [69]. A previous study of the validity and reliability of the Greek version of the questionnaire demonstrated adequate psychometric properties of the inventory for Greece’s athletic population [70]. Although EAT-26 questionnaire is not a diagnostic tool for eating disorders, it has been found to be particularly effective in detecting possible cases of eating disorders, suggesting further investigation for those with a score equal to or higher than 20. In this study, Cronbach’s α value for EAT-26 was 0.77.

#### 2.3.2. Weight Pressures in Sport-Females

The “Weight Pressures in Sport-Females (WPS-F)” Questionnaire [54] is an 11-item self-report questionnaire designed to assess the pressures that come from the sport environment and are experienced by female athletes regarding their weight, body shape, size and appearance. The answers are given on a six-point Likert scale from 1 (never) to 6 (always). The items in the WPS-F produce two factors: “Pressures from Coaches and Sports about Weight” (six items) and “Pressures regarding Appearance and Performance” (five items). The inventory has been translated into Greek and validated in Greece’s female athletic population [71]. In this study, Cronbach’s α value for the total score of the questionnaire was 0.86, for “Pressures for Coaches and Sports about Weight” it was 0.85 and for “Pressures regarding Appearance and Performance” it was 0.78.

#### 2.3.3. Social Desirability Scale

The social desirability scale (SDS) [72] is a tool consisting of 13 items that is used to examine how social desirability influences self-reported questionnaire responses. A true or false answer scale is used for each item. Eight of the items get 0 points for answering “true” and 1 point for answering “false”, while the other five items get 1 point for answering “true” and 0 points for answering “false”. The total score can range from 0 to 13, with a score of more than 9 suggesting that the responses are more likely to reflect what is thought to be socially desirable rather than the reality. The Greek version of the scale, which is translated and validated by Psychountaki et al., was used in this investigation [73].

#### 2.3.4. Other Information 

The questionnaire included a section with questions regarding the gymnastics discipline, the competition level, the years of training experience, the weekly frequency of training, the duration of training sessions and the yearly competition or event frequency (Table 1).

#### 2.3.5. Anthropometric Indices

Each participant self-reported her body weight and height, and Body Mass Index (BMI) was calculated for each participant as body weight (kg), divided by height (m^2^).

### 2.4. Statistical Analyses 

Continuous variables are presented as mean ± standard deviation and categorical variables are presented as frequencies and percentages. Chi-square tests were performed to examine differences between categorical variables. Pearson’s correlation coefficient (*r*) was used for investigating linear associations among the examined variables. Independent samples’ *t*-tests were used to examine differences in anthropometric characteristics and in the questionnaires’ scores between competitive and non-competitive athletes. For pairwise comparisons, Cohen (*d*) effect sizes were calculated, and their magnitude was categorized as follows: trivial, <0.2; small, 0.2 to 0.5; small to moderate, 0.5 to 0.8, and large, >0.8 [74].

Finally, multiple regression analyses were used to investigate the contribution of the WPS-F sub-scales to the EAT-26 score for each group of athletes. BMI was also included in the multiple regression analyses, as significant correlations were found between BMI and EAT-26 total score in the competitive gymnasts. Missing values for each parameter were noted and participants were excluded from the sample. Analyses were performed using SPSS version 22.0 (SPSS Inc., Chicago, IL, USA). For all analyses, the level of statistical significance was set at 0.05.

## 3. Results

### 3.1. Population Characteristics

**Figure 1:**
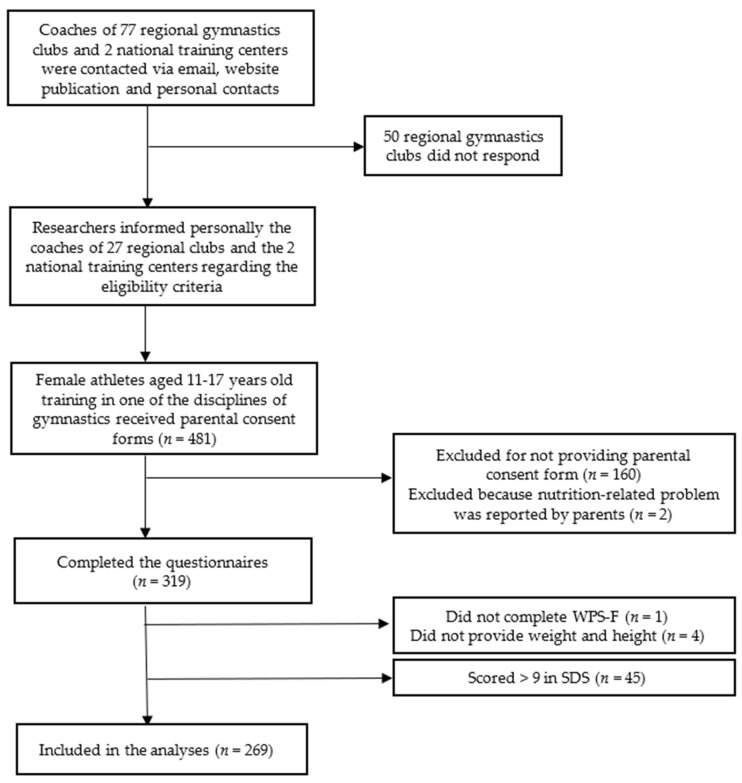
Flowchart showing the selection of the study population.

The total number of eligible female athletes aged 11–17 years old, from the 27 gymnastics clubs and the 2 national centers was 481. All participants were provided a parents’ consent form. One hundred and sixty athletes did not return their parent’s consent forms and were excluded, resulting in a total participants’ number of 321 and a participation rate of 66.7%. Moreover, two athletes were excluded because a nutrition-related problem was reported by their parents. Therefore, a total of 319 gymnasts completed the questionnaires. Forty-five athletes scored over 9 in the SDS, and were excluded from the analyses. Moreover, four athletes were excluded because they did not provide data on their weight and height, and one athlete was excluded for not completing the WPS-F questionnaire. Thus, the total number of gymnasts who were included in the analysis was 269 (Figure 1). The total sample included 50 athletes of artistic gymnastics, 42 from rhythmic gymnastics, 66 from trampoline and tumbling, 22 from aerobics and acrobatics and 89 from gymnastics for all.

One hundred and forty-seven gymnasts were competing at international and national level and were included in the competitive group. Thirty-three athletes declared participation only in local and regional events and 89 were training in gymnastics for all, and they were merged in the non-competitive group, with a total of 122 athletes. Characteristics regarding the training experience, training frequency, duration of training sessions and competition frequency are presented in Table 1.

The anthropometric characteristics of the participants are presented in Table 2. No significant differences were found between competitive and non-competitive athletes regarding their age (*p* = 0.949), but competitive gymnasts had lower height (*p* = 0.017), weight (*p* = 0.001) and BMI (*p* = 0.004) than non-competitive gymnasts.

### 3.2. Eating Disorder Symptoms and Weight Pressures

A total of 33 of the 269 athletes (12.3%) scored ≥20 in EAT-26. Regarding the different competition level groups, it was found that 24 competitive gymnasts (16.3%) and nine non-competitive gymnasts (7.4%) scored ≥20 in EAT-26. The difference between the two groups was statistically significant (Χ^2^_(1)_ = 4.961, *p* = 0.026). Moreover, competitive gymnasts scored higher than non-competitive gymnasts in the total score of EAT-26 (*p* = 0.027) (Table 3). Regarding the three sub-scales of EAT-26, no significant differences between the two groups were found. Competitive gymnasts scored higher than non-competitive athletes in the total and the two sub-scales of WPS-F (*p* ≤ 0.001) (Table 3).

In the competitive level group, positive correlations were found between the total score in EAT-26 and the total WPS-F score (r = 0.403, *p* ≤ 0.01), the sport and coaches’ weight pressures sub-scale (r = 0.353, *p* ≤ 0.01) and the appearance and performance weight pressures sub-scale (r = 0.351, *p* ≤ 0.01), while there was a negative correlation between the total score in EAT-26 and BMI (r = −0.186, *p* = 0.024). Accordingly, in the non-competitive level group, there were positive correlations between the total score in EAT-26 and the total WPS-F score (r = 0.362, *p* ≤ 0.01), the sport and coaches’ weight pressures sub-scale (r = 0.204, *p* = 0.024) and the appearance and performance weight pressures sub-scale (r = 0.412, *p* ≤ 0.01), but there was no correlation with BMI (*p* = 0.991).

### 3.3. Regression Analyses

Multiple regression analyses (stepwise forward regression) were performed separately in the two athletes’ groups to predict EAT-26 score using WPS-F sub-scales’ scores and BMI as independent variables. In the group of competitive gymnasts, the sport and coaches’ weight pressures sub-scale, the appearance and performance weight pressures sub-scale and the BMI accounted for 30.3% of the variance in EAT-26 (adjusted R^2^ = 0.303, F_(3,143)_ = 22.165, *p* < 0.001) (Table 4). The regression demonstrated that each one-unit increase in the sport and coaches’ weight pressures sub-scale corresponded to an increase of 1.510 in EAT-26 score, each one-unit increase in the appearance and performance weight pressures sub-scale corresponded to an increase of 2.954 in EAT-26 score, and each one-unit increase in BMI corresponded to a decrease of 1.554 in EAT-26 score (Table 4).

Ιn the group of non-competitive athletes, there was no correlation between EAT-26 and BMI, while the sport and coaches’ weight pressures sub-scale was not a significant factor. Therefore, the model included only the appearance and performance weight pressures sub-scale, which accounted for 16.3% of the variance in EAT-26 (adjusted R^2^ = 0.163, F_(1,120)_ = 24.481, *p* < 0.001). The regression demonstrated that each one-unit increase in the appearance and performance weight pressures sub-scale corresponded to an increase of 2.523 in EAT-26 score (Table 5).

## 4. Discussion

The most obvious findings that emerge from this study are that competitive female adolescent gymnasts present higher eating disorder symptomatology, as well as higher sport-related weight pressures, than their non-competitive peers. Moreover, it was found that weight pressures from the sport environment are associated with the development of ED symptoms. In competitive athletes, weight pressures that come from the nature of the sport and from the coaches, along with the pressures that are related to appearance, performance and BMI contribute to the risk of ED. Nevertheless, it was found that in non-competitive athletes only appearance and performance-related weight pressures contribute significantly to ED risk.

### 4.1. Prevalence of Eating Disorder Symptoms

More than 1 in 10 female adolescent gymnasts in the present study presented ED symptoms. ED symptoms were more prevalent among competitive athletes than non-competitive athletes and, as expected, competitive gymnasts scored higher in the total score of EAT-26 compared to non-competitive gymnasts. The prevalence of 12.3% of ED symptoms in the total sample is in accordance with the findings of a British study in artistic, rhythmic and acrobatic gymnasts [29] and other studies in adolescent female athletes of various sports [28,75,76]. In the group of competitive gymnasts, ED symptoms were present in the 16.3% of the sample, a percentage similar to the results of previous studies in elite artistic and rhythmic gymnasts [14,30]. Previous research in Greek elite gymnasts reported higher ED symptomatology [31,32], although it is worth pointing out that only athletes from artistic and rhythmic gymnastics were included in those studies, and/or athletes of the competitive group were all competing at international level. On the contrary, in the present study the competitive group included international and national level gymnasts from different disciplines.

Nevertheless, the finding of higher ED symptomatology in the competitive compared to the non-competitive group is in accordance with the studies of Kong and Harris (2015) and Francisco et al. (2013), which found higher ED symptomatology in elite female athletes than in recreational and non-competitive athletes [45,46]. The results are also in correspondence with the outcomes of Donti et al. (2021), which found higher prevalence of eating disorder symptoms in international rather than recreational-level rhythmic gymnasts [32].

### 4.2. Weight Pressures between Competitive and Non-Competitive Gymnasts

Moreover, the current study found significant differences between competitive and non-competitive gymnasts regarding the perceived sport environment weight pressures, with the group of competitive athletes showing higher scores in the total scale of weight pressures, as well as in the two weight pressures sub-scales.

Previous research has shown that weight-related pressures from the sport environment increase as the competition level increases [49,77]. As Teixidor–Batlle et al. (2021) indicated in a study of Spanish athletes of various sports, female athletes competing at international and national level scored higher than regional-level athletes in the teammate/uniform pressure scores and international-level female athletes scored higher on coach/sport pressure sub-scale, when compared to national and regional-level athletes [49].

### 4.3. Associations between Weight-Pressures and Eating Disorder Symptoms

The positive association that was found in the present study between weight pressures from the sport environment and eating disorder symptomatology is an interesting finding, as this was the first study in Greece that examined the sport-specific pressures that female athletes experience regarding their weight, body shape and appearance. The issue has been studied internationally, especially in “weight-sensitive” or “lean” sports, and it has been indicated that pressures from the sport environment for achieving and maintaining a low body weight or lean shape are associated with a higher risk of development of ED symptoms [17,47,51,55]. Moreover, it has been found that female athletes with ED symptoms score more highly in the weight pressure scale than athletes without ED [49].

In the current study, regression analyses indicated that the two weight pressures sub-scales and the BMI predict 30.3% of the variance in EAT-26 in the group of competitive gymnasts. However, in the group of non-competitive gymnasts, BMI and sport and coaches’ weight pressures were not significant predictors of EAT-26 score. Previous research has indicated that coaches’ perception and comments regarding weight and body shape significantly affect the presence of disordered eating behaviors in female athletes [54,78]. Nevertheless, in the sport of gymnastics, research has shown that gymnasts who are subjected to unfavorable comments regarding their weight or instructions to lose weight are more likely to develop eating disorders [15,45,55,56,79]. Coaches’ comments and demands for low body weight are expected to be lower in non-competitive gymnasts, and this may explain the finding that weight pressures from sport and coaches was not a significant factor in the non-competitive group.

On the other hand, weight pressures related to performance and appearance were found to be positively associated with ED symptoms in all competition-level groups. In the sport of gymnastics, low weight has been considered as a performance facilitator, and there is the general perception that female gymnasts should maintain a low weight to perform better [5,8,52]. Previous research in athletes of aesthetic sports indicates that if athletes believe that weight control can help them improve their sports performance, they are more likely to develop ED [17,59,60]. Moreover, performance and appearance weight-pressures include the perceived pressure from the training or competition uniform, a factor that is believed to be associated with feelings of shame and anxiety, as well as negative body image [54]. Studies in female cheerleaders, swimmers and dancers have indicated that a revealing competition uniform may make athletes feel that their perceived bodily flaws are readily apparent to spectators or judges [59,61,62,63]. Female gymnasts of all types and levels wear tight and/or revealing uniforms during their competitions, events and trainings, which may exaggerate an adolescent girl’s already-present feeling of body dissatisfaction. This may be a possible explanation of the finding that even non-competitive gymnasts are feeling pressured regarding their weight and shape, but the stronger pressures are those that are related to appearance.

Finally, one of the study’s more interesting findings was that BMI was a moderator in the correlation between weight pressures and ED symptoms, but only in competitive gymnasts. Regression analyses revealed that there is a negative association between BMI and EAT-26, showing that a higher BMI predicts lower EAT-26 score. This finding should be interpreted with caution, as it is known that low BMI could be a symptom of eating disorders, but in the current cross-sectional study a causal relationship could not be established. Nevertheless, this finding is consistent with a recent Norwegian study of adolescent athletes that found a higher incidence of disordered eating in athletes of individual sports who had low BMI [80]. On the other hand, in a study of adolescent female swimmers in Brazil, athletes with disordered eating were found to be heavier than athletes without disordered eating [81], while a study of adolescent female athletes in Slovenia revealed that the risk of developing ED was associated with a higher BMI and fat percentage [82]. It is interesting that the negative correlation between BMI and EAT-26 wasn’t present in non-competitive athletes. This finding, in combination with the previous one that sport and coaches’ weight pressures do not contribute to the development of ED in non-competitive gymnasts, could indicate that, in athletes who do not have high competition requirements, development of ED is associated to a greater extent with factors outside the sport environment. As it was found by Francisco et al. (2013), social pressure was the most significant predictor of disordered eating in non-elite young aesthetic athletes [46].

### 4.4. Limitations

This research has some limitations that should be noted. First, the study’s cross-sectional design makes it impossible to demonstrate a causal relationship between the variables studied. Second, self-reported questionnaires were used for data collection, which may have increased the risk of producing socially acceptable responses. To reduce this risk, the SDS was used to exclude athletes who gave answers that were judged as “socially desirable” rather than accurate.

Furthermore, in this study, an eating disorder diagnostic assessment was not performed. Gymnastics sports have a high prevalence of disordered eating behaviors and it is possible that some athletes are unaware of their symptoms or choose not to report them. This is a serious constraint that we attempted to overcome by ensuring that the athletes’ answers were anonymous and confidential and by asking parents/guardians (via the consent form) to report any nutrition-related condition (such as diabetes, celiac disease, or anorexia nervosa) of their child. Another limitation that should be noted is that it was chosen to include athletes from different disciplines of gymnastics and not only by the more widely studied disciplines of artistic and rhythmic gymnastics. This decision was made in order to include a greater number of adolescent female gymnasts in the study, taking into account the fact that in Greece all different disciplines of gymnastics are popular among adolescent females. However, it is a common belief that artistic and rhythmic gymnastics are considered disciplines with higher training demands and weight pressures than the rest of the disciplines. Moreover, in this study only female gymnasts participated.

Finally, it should be noted that athletes self-reported their anthropometric characteristics (height and weight) and no other anthropometric measurements were performed. As a result, there is the possibility that athletes under- or over-reported their weight or height, a fact that may have affected the association of BMI with EAT-26.

### 4.5. Strengths of the Study

To our best knowledge the present study is the first conducted in Greece that examines the sport-related weight pressures regarding athletes’ body weight, shape and appearance and the possible associations with eating disorder symptoms. Moreover, it is the first study in Greece in which gymnasts from different competition levels of all the gymnastics disciplines participated. The included sample size is much larger than that of previous studies among Greek gymnastics athletes. Finally, the study included female adolescent gymnasts from many different geographical areas of the country.

## 5. Conclusions

The current study indicated that a significant number of adolescent gymnasts of all disciplines and competitive and non-competitive levels in Greece present ED symptomatology. National and international level athletes seem to be at greater risk of developing eating disorders and this finding highlights the need for providing timely diagnosis and treatment in this vulnerable population. A factor that significantly increases the risk of developing eating disorder symptoms is the pressures that come from the sport environment regarding the achievement of a low weight and a lean shape. This finding was documented in gymnasts of all competition levels.

The results of the present study could be used for implementing prevention measures regarding disordered eating attitudes in the sport of gymnastics and especially in elite athletes. Interventions should aim to improve the awareness of coaches, judges and sports officials regarding the impact that low-weight pressures have on the physical and psychological health of young gymnasts.

Low but healthy weight is an important factor for gymnastics performance. Therefore, and in order to minimize the use of unhealthy weight control measures, all adolescent gymnasts should be offered body composition and nutritional assessment by a registered dietitian. In case that body composition changes are required, the dietitian should provide individualized nutritional intervention in order for the gymnast to achieve optimal body weight and body composition through balanced nutrition. Coaches should avoid giving nutritional advice and they should refer to dietitians instead. Referral to dietitians for individualized nutritional counseling for every athlete that needs it is suggested by the American College of Sports Medicine [83]. Moreover, the need for nutritional counseling for adolescent athletes has been thoroughly mentioned, as this population group is considered nutritionally vulnerable due to both growth and training demands [40,84]. A registered dietitian could also diagnose disordered eating attitudes at an early stage and could advise athletes on how to maintain a healthy weight while avoiding the negative consequences of unhealthy dieting practices. Counseling from mental health experts is also crucial in helping athletes feel confident regarding their body appearance and their performance capabilities, as well as giving psychological counseling to those who present symptoms of eating disorders. It is a common belief that all adolescent gymnasts should be able to enjoy their sport without feelings of fear, guilt and anxiety regarding their body weight and body shape.

Future research in adolescent gymnasts could include males, too. Factors that were not included in the present study, such as the influence of family and social media, should also be addressed. Perceptions of the coaches regarding ideal weight, dieting practices and their attitudes towards athletes could also be included in future investigations.

## Figures and Tables

**Table 1:** Training characteristics of the participants (frequencies and percentages) (*N* = 269).

		Competitive (*n* = 147)	Non-Competitive (*n* = 122)	X^2^	*p*
Training Experience (years)	>8 years	47 (32.2%)	25 (20.7%)	12.319	0.002
	6–8 years	60 (41.1%)	39 (32.2%)		
	<6 years	39 (26.7%)	57 (47.1%)		
Training frequency (sessions/week)	≥5 sessions/week	120 (81.6%)	14 (11.6%)	133.105	<0.001
	3–4 sessions/week	23 (15.6%)	69 (57.0%)		
	1–2 sessions/week	4 (2.7%)	38 (31.4%)		
Training session duration (hours/session)	>4 h	36 (24.5%)	0 (0.0%)	118.908	<0.001
	3–4 h	81 (55.1%)	16 (13.3%)		
	1–2 h	30 (20.4%)	104 (86.7%)		
Competitions/Events Frequency (events/year)	>3/year	51 (35.2%)	26 (21.8%)	35.326	<0.001
	2–3/year	82 (56.6%)	47 (39.5%)		
	1/year	12 (8.3%)	46 (38.7%)		

**Table 2:** Participants’ age and anthropometric characteristics (mean ± SD) (*N* = 269).

	Competitive (*n* = 147)	Non-Competitive (*n* = 122)	t (df = 267)	*p*	Cohen’s *d*
Age (years)	13.89 ± 1.75	13.91 ± 1.76	0.064	0.949	0.01
Height (cm)	156.32 ± 8.72	158.78 ± 7.83	2.409	0.017	0.29
Body weight (kg)	45.93 ± 8.87	49.37 ± 8.48	3.223	0.001	0.39
BMI (kg/m^2^)	18.63 ± 2.32	19.49 ± 2.54	2.890	0.004	0.35

BMI: Body Mass Index.

**Table 3:** Scores (mean ± SD) of the participants in the items of the inventories (*N* = 269).

	Competitive (*n* = 147)	Non-Competitive (*n* = 122)	t	*p*	Cohen’s *d*
EAT-26	10.99 ± 8.08	8.91 ± 6.95	2.230	0.027	0.27
Dieting	6.36 ± 6.03	5.06 ± 5.72	1.804	0.072	0.22
Bulimia and Food Preoccupation	0.74 ± 1.40	0.56 ± 1.19	1.089	0.277	0.14
Oral Control	3.79 ± 3.39	3.24 ± 2.77	1.457	0.146	0.18
WPS-F	3.23 ± 1.04	2.43 ± 0.95	6.562	0.000	0.80
Pressures from Coaches and Sports about Weight	3.50 ± 1.16	2.46 ± 1.17	7.285	0.000	0.89
Pressures regarding Appearance and Performance	2.91 ± 1.23	2.39 ± 1.13	3.568	0.000	0.44

**Table 4:** Multiple regression analysis predicting EAT-26 among competitive level gymnasts (*N* = 147).

						95% CI	
Variables	B	SE	Βeta	*t*	*p*	Lower	Upper
Pressures from Coaches and Sports about Weight	1.510	0.563	0.217	2.681	0.008	0.397	2.624
Pressures regarding Appearance and Performance	2.954	0.589	0.449	5.012	0.000	1.789	4.119
BMI	−1.554	0.273	−0.447	−5.692	0.000	−2.094	−1.014
	Adjusted R^2^ = 0.303, *p* < 0.001						

CI, confidence intervals; SE, standard error.

**Table 5:** Multiple regression analysis predicting EAT-26 among non-competitive level gymnasts (*N* = 122).

						95% CI	
Variables	B	SE	Βeta	*t*	*p*	Lower	Upper
Pressures regarding Appearance and Performance	2.523	0.510	0.412	4.948	0.000	1.514	3.533
	Adjusted R^2^ = 0.163, *p* < 0.001						

CI, confidence intervals; SE, standard error.

## Data Availability

All data are available upon request to the first author.
